# A Novel Energy Replenishment Algorithm to Increase the Network Performance of Rechargeable Wireless Sensor Networks

**DOI:** 10.3390/s24237491

**Published:** 2024-11-24

**Authors:** Tariq, Vishwanath Eswarakrishnan, Adil Hussain, Zhu Wei, Muhammad Uzair

**Affiliations:** 1 School of Electronics and Control Engineering, Chang’an University, Xi’an 710000, China; 2021032905@chd.edu.cn (T.); 2022032907@chd.edu.cn (A.H.); wzhu@chd.edu.cn (Z.W.); 2 Meta Platforms Inc., 1 Hacker Way, Menlo Park, CA 94025, USA; 3 Department of Electronic Engineering, International Islamic University, Islamabad 44000, Pakistan; uzairmuhammad501@gmail.com

**Keywords:** wireless recharging, mobile charger, sensor network, WSN

## Abstract

The emerging wireless energy transfer technology enables sensor nodes to maintain perpetual operation. However, maximizing the network performance while preserving short charging delay is a great challenge. In this work, a Wireless Mobile Charger (MC) and a directional charger (DC) were deployed to transmit wireless energy to the sensor node to improve the network’s throughput. To the best of our knowledge, this is the first work to optimize the data sensing rate and charging delay by the joint scheduling of an MC and a DC. We proved we could transmit maximum energy to each sensor node to obtain our optimization objective. In our proposed work, a DC selected a total horizon of 360° and then selected the horizon of each specific $90^{\circ }$ area based on its antenna orientation. The DC’s orientation was scheduled for each time slot. Furthermore, multiple MCs were used to transmit energy for sensor nodes that could not be covered by the DC. We divided the rechargeable wireless sensor network into several zones via a Voronoi diagram. We deployed a static DC and one MC charging location in each zone to provide wireless charging service jointly. We obtained the optimal charging locations of the MCs in each zone by solving Mix Integral Programming for energy transmission. The optimization objective of our proposed research was to sense maximum data from each sensor node with the help of maximum energy. The lifetime of each sensor network could increase, and the end delay could be maximized, with joint energy transmission. Extensive simulation results demonstrated that our RWSNs were designed to significantly improve network lifetime over the baseline method.

## 1. Introduction

There are many applications for wireless sensor networks (WSNs), such as environmental detection, civil infrastructure, target tracking, information security, and intelligent media care [1,2]. In WSNs, energy replenishment is becoming the bottleneck to improving network utility. Currently, most sensor nodes are powered by batteries. However, the high manufacturing and swapping costs of batteries hinder WSNs from fully realizing their potential. Wireless charging is one of the most promising technologies for tackling the energy limitation problem of sensor nodes. A sensor node’s lifespan can be extended without battery replacement with the use of a wireless charger. The sensor node’s capabilities include sensing, computation, data gathering, and data aggregation [3,4]. The sensor node transmits data to a sink node in real time and receives control instructions from a cloud server using a wireless connection module. As sensor nodes analyze and send substantial information, the energy of the RWSN progressively diminishes over time [5]. When the battery energy gets low and cannot be restored [6], this results in the breakdown of the sensor nodes. Consequently, the energy consumption of sensor nodes has emerged as a critical concern and is considered a primary determinant in the large-scale implementation of RWSNs. To enhance the longevity of RWSNs, scheduling algorithms for sensor nodes are presented to optimize energy utilization. Most rechargeable batteries are nonremovable, resulting in sensor nodes being inoperative because of battery energy depletion.

Recent studies [7,8] have proposed using mobile chargers to transmit wireless charging power to sensor nodes [9]. The weakness of previous studies is that mobile chargers cannot transmit proper energy because they have limited battery capacity and select limited routing paths to transmit charging power to the area of the RWSN. Different sensor nodes cannot maintain their working power because of the limited energy. In our proposed research, a mobile charger and a directional charger were used jointly to transmit wireless charging power to each sensor node to overcome these challenges. To the best of our knowledge, we transmitted maximum energy to sense maximum data from the maximum number of sensor nodes. The predetermined recharging scheduling of mobile chargers provided a near-optimal charging path because of the chargers’ dynamic energy consumption. In RWSNs, a directional charger transmits energy to sensor nodes from different orientations. Each directional charger selects its total 180° area, and each orientation covers a 90° area to fulfill the energy demand of each sensor node. Directional chargers cover the network area in two directions; each orientation covers 90 areas. Mobile chargers cover the nodes that are out of the coverage area of the directional chargers. The critical challenge of our designed research was coupling directional chargers and mobile chargers to fulfill the energy demand without delay.

In our proposed research, the Mix Integral Programming method was used to find the optimal stopping point of mobile chargers. After selecting the optimal stopping point, the mobile and directional chargers jointly started energy transmission. At each time slot, the mobile and directional chargers selected their numbers of sensor nodes for energy transmission. We had to transmit maximum energy at each time slot to overcome the joint energy replenishment challenge. In the RWSNs in our proposed research, a time-slot-based algorithm was used for energy transmission to each node. The joint charging process was used to transmit energy to each set of networks. We used a priority-based charging method. In this optimal algorithm, when a node has less residual energy, the priority of energy supplement is higher, and that node should be replenished with higher charging priority. After that, we selected the tour length of the mobile charger for maximum energy transmission. Some nodes far from the mobile charger’s tour could not replenish energy properly. These sensor nodes would be charged in the next tour. In this algorithm, we needed to bind the length of the mobile charger tour to transmit maximum energy within each time slot. Moreover, we used an optimal joint-based charging algorithm to charge sensor nodes jointly with the help of a mobile charger and a directional charger.

This research aimed to optimize the data transmission rate while preserving charging delay. DCs alone cannot satisfy all the charging demands of each node in an RWSN because of a fair charging delay. To tackle this issue, we needed to schedule the MC and DCs jointly to fulfill the charging demands of each node. The MC started the route from the base station, and after transmitting energy to different sensor nodes, it returned to the base station for energy replenishment.

The main contributions of this paper can be summarized as follows:

- To the best of our knowledge, this is the first work to study the joint scheduling problem of DCs and MCs for energy replenishment. We noticed that the throughput of WRSNs can be further improved by jointly working on the performance of the MC and DCs.
- We designed a near-optimal joint energy replenishment algorithm and proposed a central charging point selection algorithm for proper energy transmission.
- Extensive simulations were conducted to indicate the effectiveness and advantages of our proposed algorithms.

The organization of this paper is as follows: Section 2 describes the related work. Section 3 contains the methodology. Section 4 presents the network model. Section 5 presents the charging model, and Section 6 presents the problem formulation. Section 7 presents the Mobile Charging Energy Transmission Protocol. Section 8 presents the evaluation and simulation results of the proposed scheme, the latter of which are compared with those of other schemes. Section 9 shows the conclusion of this paper with a summary of new findings.

## 2. Related Work

Different existing research has focused on energy harvesting techniques that extract energy from solar, vibration, and wind to replenish energy in sensor nodes. Wireless energy transfer technology provides a new way of supplying high-density, stable, and sustainable energy for sensor nodes. Recently, Powercast has developed products that transmit energy to the sensor nodes [10]. The authors of [11] used Powercast chargers to extend the network lifetime in constructing a charging queue based on the greedy algorithm.

### 2.1. Mobile Charging Scheduling

For replenishing energy to each sensor node through strong magnetic resonance, there has been a surge of interest in using the MC traveling algorithms in WRSNs [12]. Most of the reported approaches employed optimization algorithms to solve wireless charging problems. A single mobile charger can charge sufficiently a small-scale network of RWSNs. Due to the limitation of the battery capacity of the sensor nodes, multiple mobile chargers are required for large-scale areas of a network. Energy replenishment provides the stability and reliability of energy supply for sensor nodes [13]. The mobile charging energy transmission process can be evaluated from the sensor node’s and charger’s perspectives [13]. The most intuitive way is to charge sensor nodes periodically along an optimal TSP tour in RWSNs [8].

The researchers employed a mobile charger to charge sensor nodes, where the cumulative traveling time and charging time are expected to be minimized. The best charging scheme was reported [13], which jointly considers routing and charging. In a dynamic environment, the objective is to maximize network lifetime under practical constraints. In [14], the authors proposed multiple energy-constrained mobile charging schemes to charge RWSNs collaboratively. The aim was to maximize the energy efficiency of sensor nodes. In [8,15], the authors focused on a charging strategy that employed a mobile charger equipped with a high-capacity battery to replenish energy periodically in sensor nodes. In [16], the issue pertains to 2-D wireless sensor networks and the small quantity of energy-constrained mobile chargers. The objective of this endeavor is to ensure the network’s performance. The average performance is far below that of an ideal charger. In [17], the issue of reducing the quantity of mobile chargers to guarantee uninterrupted energy transfer for each sensor node was examined. The varying charging cycles of MC yield diverse outcomes for the charging tour. In [18], numerous mobile chargers were analyzed to minimize the overall trip cost of the mobile charger, ensuring that each node receives energy replenishment from the sensor node. In [19] an on-demand charging method was modeled for scheduling multi-copter vehicles assigned to charge sensor nodes. The aim was to reduce the number of mobile chargers.

In [20], the scheduling of multiple MCs for incoming charging requests assessed the nearest-first and recent-rarest-first techniques. The closest scheduling technique was superior for the localization of the charge request process, as demonstrated through simulation. In [18], the node’s energy level cannot decrease from its maintaining level. Multiple mobile chargers reduced the overall travel expenses of various mobile charging units. In [19], MCs returned to their designated depots for energy replenishment. The network model addressed on-demand charging issues for sensor nodes in WRSNs according to the schedule of a mobile charger. The aim was to reduce the number of mobile charger routes to optimize energy transfer.

### 2.2. Directional Charging Scheduling

Some works study the directional charger problem, but their solutions have not been applied in our research. In [21], the authors proposed a method to detect omnidirectional charging capability for a given topology of directional chargers. They also studied how to place directional chargers to maximize charging utility in [22] to resolve the wireless charger deployment optimization problem. In [23], chargers with directional antennas were deployed on specific grid points and designed the greedy and adaptive cone covering algorithms. To maximize the survival rate of end devices, an omnidirectional wireless charger with partial coverage has been used to maximize the survival rate of terminal devices [24].

One of the important aspects of a directional antenna is that it transmits maximum energy in different directions to cover different sensor nodes in one sector. However, these models are so complicated that they must be tractable in the performance analysis. Using directional antennas instead of omnidirectional antennas in wireless ad hoc networks or RWSNs can significantly improve the network performance since directional antennas can concentrate the transmitting/receiving capability to desired directions [25].

**Figure 1:**
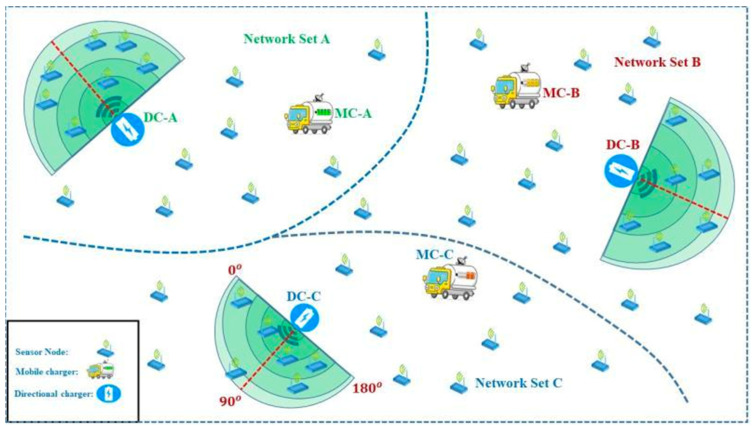
Wireless rechargeable sensor network.

Figure 1 shows the WRSN. For example, some recent studies have shown that using directional antennas in WANs can improve the network capacity and reduce the end-to-end delay [26,27,28,29]. In addition, using directional antennas in WSNs can improve security, as shown in [25,30]. In WSNs with directional antennas, it is difficult for each node to obtain the location knowledge of other neighbors due to directional beam forming [31]. To solve the problem of directional neighbor discovery, complicated schemes, such as using direction of arrival estimation, and swiveling the beam from 0 to 2π, were proposed in [32].

To the best of our knowledge, this is the first work to study the mobile charger and directional charger problem in RWSNs. The existing works have contributed to optimizing the power supply through the mobile charger and directional charger. However, coupling issue in mobile chargers and directional chargers makes the rechargeable wireless sensor network charging problem quite challenging. Previous works did not consider the joint charging method using a mobile charger and directional charger jointly to transmit maximum energy and minimum charging delay. The novelty of our proposed research is that there are no data loss in our proposed work because of on-demand energy transmission to each sensor node.

## 3. Methodology

The critical challenge of our design is the coupling of DC and MC used to fulfill the energy demand without delay. In RWSNs, each DC selects its total $360^{\circ }$ area, with each $90^{\circ }$ specific orientation to fulfill the energy demand of each node in every time slot. MC selects a network area that is out of the coverage area of DC. The charging process with the MC antenna is high gain, which is used for energy transmission in each set of networks. MC selects the optimal stopping point in each set of networks to charge all the sensor nodes at different time slots. After selecting a specific network area, MC and DC jointly start energy transmission to each sensor node. At each time slot, MC and DC select a different number of sensor nodes for energy transmission. MC travels from the base station to each charging point, which is obtained by solving Mix Integral Programming. When the energy level of MC is low, it returns to the base station for energy replenishment.

We need to choose different MC stopping points from each set of sensor nodes from each set of networks. After selecting a specific area by MC and DC, both chargers jointly start energy transmission to the sensor node. At different times, slot MC turns its antenna orientation toward the area of DC to transmit maximum energy. To overcome the joint energy replenishment challenge, we need to transmit maximum energy to each sensor node at each time slot to collect maximum data from each node. It is quite challenging to utilize the limited energy of DC and MC to charge the lowest energy demand sensor node and maintain the network performance continuously. MC selects each set’s route from the base station for energy transmission. MC finds the optimal stop location in each set of sensor nodes with the help of Mix Integral Programming. In RWSNs, after finding the optimal location, MC starts to replenish charging power to each node that is out of the DC coverage area and in the DC coverage area.

Voronoi diagram has been used to divide the network area into different sets. In RWSNs, we must allocate a time slot-based algorithm for energy transmission to each node. The joint charging process is usually used to transmit energy in each set of networks. For proper energy transmission to each node, we select the tour length of MC. We bound the length of the MC tour to maintain network operation. The proposed work’s objective is to increase each node’s sensing rate.

**Figure 2:**
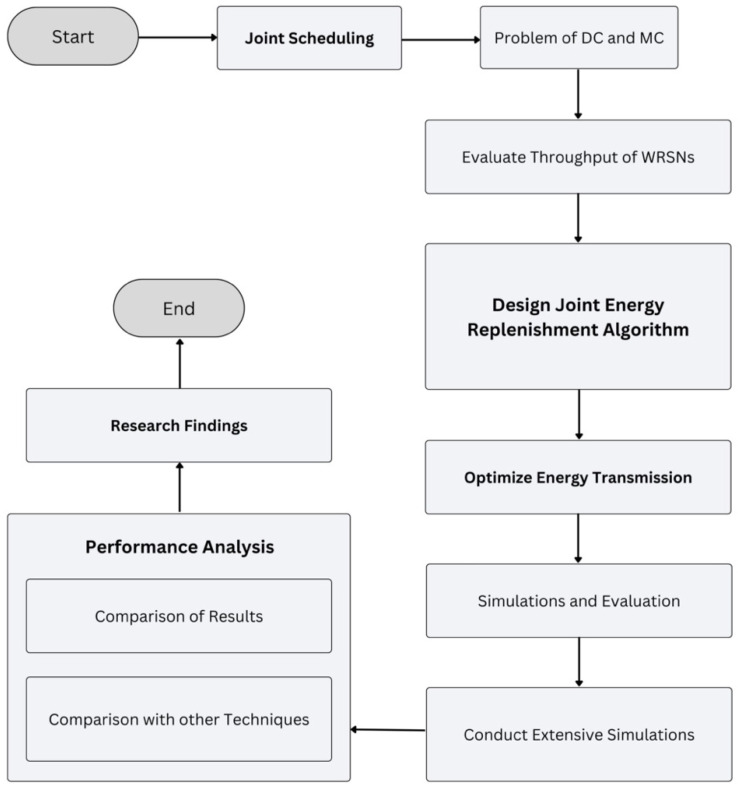
Methodology.

In our proposed research, a simulation is built in the widely used MATLAB. The experiment results show that our proposed algorithm works efficiently to obtain maximum throughput. The main contribution of this paper can be summarized as follows: To the best of our knowledge, this is the first work to study the joint scheduling problem of DC and MC for energy replenishment. We noticed that the throughput of WRSNs can be further improved by jointly working on the performance of the MC and DC. We designed a near-optimal joint energy replenishment algorithm and proposed a central charging point selection algorithm for proper energy transmission. Extensive simulation tests were conducted to indicate the effectiveness and advantages of our algorithms. Figure 2 shows the methodology of the research work.

**Figure 3:**
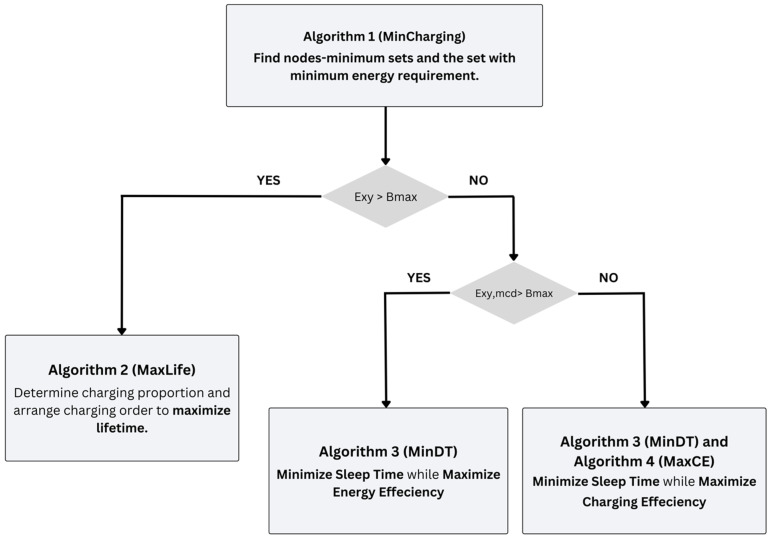
Algorithm framework.

This flowchart contains a multi-step algorithmic methodology to increase power consumption and charging productivity. It starts with the first algorithm (Min-Charging), which distinguishes the arrangement of nodes with the required minimum energy assumption. Depending on whether the energy condition $E_{xy}$ exceeds the most extreme limit of the battery, (B maximum, the cycle process into different paths. It is unlikely that $E_{xy}$ is more remarkable than the calculation of B maximum) the second algorithm (MaxLife) is used to decide on charging, and the cycle checks if $E_{xy}$, $M_{cd}$ exceeds B maximum. Assuming it does, algorithm 3 (MinDT) is used to limit rest time and increase energy productivity with the extra chance that $E_{xy}$, $M_{cd}$ is not precisely B maximum. Both calculations in algorithm 3 (MinDT) and algorithm 4 (MaxCE) are used to limit the rest time and expand the charging efficiency. This organized methodology is expected to improve the total energy of the system and the functionality efficiency of the overall system. Figure 3 shows the framework of the algorithms.

## 4. Network Model

In RWSNs, it should be desirable that all the sensor nodes should be charged before their energy depletion. To achieve this goal, we need to transmit maximum charging power to the maximum sensor node at each time slot. In RWSNs, a set of rechargeable sensor nodes $S=\left[s_{1},s_{2},s_{3},\ldots ,s_{n}\right]$; a set of sink stations $A=\left[a_{1},a_{2},a_{3},\ldots a_{n}\right]$; and a set of directional chargers $D=\left[d_{1},d_{2},d_{3}\ldots d_{n}\right]$ are deployed randomly at different positions where each sensor node senses data and transmits data to the sink node. In RWSNs, we jointly used MC and DC to transmit maximum energy to maximum sensor nodes as per energy demand. When the DC cannot satisfy the recharging delay required by the nodes due to constraints such as energy distribution or scheduling limits, using an MC in addition to a DC ensures timely and efficient energy replenishment for nodes. The MC improves the DC by delivering concentrated charging to nodes with significant energy demands, ensuring load balance and redundancy, preventing node failures, and maintaining network reliability. Additionally, the MC reduces the potential for shadowing or line-of-sight constraints that could impede the DC’s effectiveness. This mixed strategy optimizes network efficiency by allowing the DC to oversee a wide range of coverage. At the same time, the MC focuses on critical energy support, thereby guaranteeing the continuous operation and resilience of the network.

After replenishing energy from the basic station, MC selects its shortest routing path to charge sensor nodes that are out of coverage of DC as well as under the coverage of MC. At different time slots, MCs turn their route toward the DC coverage area and transmit energy to the sensor nodes in the DC coverage area to maintain each node’s energy level. Therefore, in the proposed scheme, we scheduled the routing path of MC to cover sensor nodes that are out of the coverage area of DC and those that are under the coverage area of DC.

In this research, we applied a tree topology with a fixed routing path, where each sensor node had only a single link to the next hop. One bit of data is sensed by node i and is transmitted to the sink station through a fixed routing table. We divided nodes into different sets. In RWSNs, we must schedule the MC to transmit maximum energy to each sensor node.

We divided the time series of DC into different slots $T=\left[t\ldots ,t\right]$. In each time slot, DC changes its orientation from two orientations with $90^{\circ }$ to transmit charging power to the different no-node in the area of the network. At each orientation, the directional charger covers a different number of nodes with a $90^{\circ }$ angle. DC covers each orientation with a $90^{\circ }$ angle to increase the coverage length of the nodes for energy transmission. The total coverage area of DC is a $180^{\circ }$ angle. MC M covers the remaining 180 angle area which the DC uncovers.

In RWSNs, each node has a different energy consumption rate at different times. Each sensor’s energy consumption rate varies and is impacted by several factors. This fluctuation may be related to event-driven sensing, as sensors consume more energy during increased activity than idle times. Moreover, sensors performing various activities require differing energy levels. Besides the sensor’s location concerning energy sources like MCs or DCs, energy consumption may also be affected by variable data transmission demands and adaptive duty cycles that change according to event likelihood or power conservation needs. We need to select an optimal stopping point of MC from each set of sensor nodes to transmit maximum charging power to maximum sensor nodes. This research aims to maximize network lifetime with the help of maximum energy transmission. Thus, we apply the following model for optimization.

(1) $$MAX\;r_{C_{ij}}x_{ij}=+\Sigma_{t=1}^{T}\Sigma_{i=1}^{N}$$

(2) $$s.t.\;r_{i}+f_{it}^{\left(in\right)}=f_{it}^{\left(out\right)}$$

(3) $$e_{o}\left(i\right)r_{i}+e_{r}\left(i\right)f_{i}^{\left(in\right)}+e_{t}\left(i\right)f_{i}^{\left(out\right)}\geq e_{c}\left(i\right)$$

(4) $$e_{c}\left(i\right)=\Sigma_{i=1}^{M}X_{ij}P_{c}\left(ij\right)$$

(5) $$X_{ij}=C_{ij}Y_{j}$$

Equation (1) shows the sensing rate of node i, and Equation (2) shows the data conservation constraint. For each sensor node, the aggregated incoming data flow equals the aggregated data outgoing flow. Equation (3) calculates the available charging power of every sensor node. Equation (4) is the power which shows that the consumption charging power should never exceed the total charging power. Equation (5) shows the sensor node coverage area in the network area where the MC transmits charging power at the node. Table 1 provides the notations.

## 5. Charging Model

Our design is based on the Powercast wireless charging and sensing platform. We utilized the Friis space equation, which has been experimentally proven to be a good approximation of the energy recharging and commonly applied, to represent the energy recharging model in Equation (6). We claim that our design can work on any energy-recharging model whose charging power is monotone and increases with the distance between the charger and sensor node.

(6) $$Pr=\frac{GsGr\eta }{Lp}\left(\frac{\lambda }{4\pi {\left(d+b\right)}^{2}}\right)p_{o}$$

where d is the Euclidean distance between the charger and sensor node. $p_{o}$ is the source of power. $G_{s}$ is the source antenna gain. $G_{r}$ is the receive antenna gain. $L_{p}$ is the polarization loss. η is a parameter to adjust the model for short-distance transmission. λ is the wavelength. b is the parameter to set the rectifier efficiency. We simplify the energy charging model as shown in Equation (7) for easy presentation.

(7) $$P=\frac{a}{{\left(d+b\right)}^{2}}$$

## 6. Problem Formulation

In our proposed scheme for WRSNs, we first divide the network area into different sets for proper energy transmission to each sensor node. With the help of different sets, we can transmit maximum energy to each sensor node. We used a Voronoi-based algorithm to divide the network area into different sets. In each network set, one DC and MC are used to transmit energy to the sensor nodes in RWSNs. The process of charging with a mobile charger is highly gained. We can combine nodes within different sets N, and then all the nodes in the coverage of DC and out of the DC coverage area in G can be charged at different time slots.

### 6.1. Time Slot-Based Charging

The proposed charging algorithm jointly works with directional and mobile chargers. The schedule of directional chargers and mobile chargers is identical and the charging activities of each set of nodes are repeated at different times. Due to the time-varying nature of energy demand, we divide the time for joint energy transmission in each node into fixed time intervals with length L. MC contains multiple slots for energy transmission. In each time slot, MC runs a tour to selectively recharge sensor nodes from low energy levels. The energy transmission of one node should be finished before the current slot ends to ensure that the next node energy transmission can be timely started in the next period. Sensor nodes and DC consume energy at different rates in the network area. We need to divide the time for energy transmission according to slot length. MC and DC jointly recharge sensor nodes to maintain the energy level of each node. A slot is the smallest time unit where one charger can replenish energy to each node. In the network area, MC is responsible for energy transmission at each set of network areas. In each set of networks, time is divided for different sensor nodes and directional chargers. Each set has one DC which covers sensor nodes. At each time slot, the MC antenna changes its orientation to select a node for energy transmission.

The length of the slot is $T=\left(a-\right)T_{m}$ which is the duration of each node for energy transmission. The required charging period of each node is based on slot length. The cycle length L is the required charging period of all the sensors in each set of networks. The duration of the charging period of each node can be counted in terms of the slot. Suppose the sensor is categorized into the number of $N=\left(n_{1},n_{2},\ldots ,n_{n}\right)$ in set. The required charging period of each set is $T=\left[t_{1},t_{2},\ldots ,t_{n}\right]$ in each slot.

$M=\left[m_{1},m_{2},\ldots ,m_{n}\right]$ with each node set and slot-based design. Based on the node set, we can periodically schedule the power replenishment into slots. According to the schedule, MC selectively charges a set of nodes in each period. We can give slots based on periodical charging. We first obtain nodes with equal energy demand and then finally obtain recharging nodes as they have low data sensing rates because of low energy levels. The charging tasks are issued in different slots at each time. A slot is called a working slot once the charging task issued in the slot. This algorithm aims to obtain all the working slots in one stopping point of MC.

### 6.2. Optimal Stopping Point with Mix Integral Programming

In a RWSN, the feasible region is the region where all the constraints are satisfied. Pseudo-code for the feasible region is as B is the base station in the RWSN area. $R_{i}$ is the radius, n is the number of nodes, and N in one set of network areas. Assume all the nodes use power $p_{o}$.

The objective is to refine the continual cessation of the charger inside each network region, with target node A identified by the circle encompassing the charger’s two random stopping positions, $x_{1}$ and $x_{2}$. Let $d=\left(x_{1},x_{2}\right)$ represent the Euclidean distance. $t=\left(i,x\right)$ denotes the charging time for node i when the charger halts at point x. If $t=\left(i,x_{1}\right),\leq t=\left(i,x_{2}\right)$ then $d=\left(i,x_{1}\right)\leq d=\left(i,x_{2}\right)$ with all the viable areas remaining as convex polygons. The ideal place or location for a mobile charger typically occurs in the corner of the feasible zone.

The feasible zone is defined as $p_{i}$ representing the energy available at source *i*, where $i=\left(1,2,3\ldots n\right).$ $p_{j}$ represents the energy accessible at destination j as $j=\left(1,2,3,\ldots n\right).$ $X_{ij}$ represents energy distribution from source node i to destination node j. $C_{ij}$ represents the energy loss from source node i to destination j. The problem may be formally addressed by mixed integer programming as shown in Equations (8)–(11).

(8) $$\;\Sigma_{i=1}^{m}\Sigma_{j=1}^{n}C_{ij}X_{ij}$$

(9) $$\;\Sigma_{i=1}^{m}\Sigma_{j=1}^{n}=p_{i}\left(i=1,2,3,\ldots n\right)$$

(10) $$\;\Sigma_{i=1}^{m}\Sigma_{j=1}^{n}\left(X_{ij}\right)=e_{i}\left(i=1,2,3,\ldots n\right)$$

(11) $$\;x_{ij}\geq 0$$

Now consider the pair of nodes A and B as the targeted node and base node with feasible regions with $R_{2}$ and $R_{1}$ in each set of networks. Let the charger stop at $x_{r},$ then Equations (12) and (13) are as follows

(12) $$\;\forall x_{A}\epsilon R_{2}$$

(13) $$\;d\left(x_{A},x_{r}\right)<d_{m}ax\left(R_{1},x_{r}\right)$$

and Equations (14) and (15) are

(14) $$\;\forall x_{A}\epsilon R_{1}$$

(15) $$\;d\left(x_{B},x_{r}\right)<d_{min}\left(R_{2},x_{r}\right)$$

## 7. Mobile Charging Energy Transmission Protocol

The process of charging with a mobile charger antenna is highly gained which is usually used to transmit energy. We can combine nodes within different sets N and each set has a directional charger. When MC stops at the center of N then all the nodes and one directional charger in G area can be charged at different time slots. There are different nodes and one DC in each set of network coverage areas which is covered by the mobile charger. The real number of networks cannot be larger than N.

G is the region and can be obtained from one set of network areas that $G=\pi^{2}tan^{2}\left(\theta^{2}\right)$ where d is the shortest distance between MC and DC. The charging angle θ is for MC to cover the set of nodes and DC. In the G area, to satisfy DC with charging power, we assign the maximum power consumption rate of all the nodes as well as DC in G area which is $p_{G}=max\left(p_{i}\epsilon G\right)$.

To ensure that the maximum number of nodes and DC to meet the relation n<u/p in which u is the charging power and p is the average power consumption in all the DC nodes and the coverage area. We set the average charging time t of the mobile charger to ensure that the total charging power is greater than or equal to the energy depletion rate of all the sensor nodes and DC, that is, Ut−npt≥0. By the setting of t and performing transposition, we obtain n≤Up. Due to the existence of mobile charger moving time $t_{m}$, it would lead to $U\cdot t-np\left(t+t_{m}\right)>0$. As long as $t_{m}$ exists, even very small, the relation can be obtained as U−np>0, that is n<u/p⋅s.

We define the MC charging coverage utility at stop $s_{k}$ on MC charging orientation $\theta_{sk}$ as the sum of the received power of the charging.

$\left(s_{k},\theta_{s}k\right)$ denotes the coverage area that is covered at the one-stop of MC in any MC antenna orientation $s_{k}$. MC stopping point $s_{k}$ is in charging orientation $\theta_{s}k$ and MC stops at the m point and finds optimal antenna orientation $\left\{\theta_{s}k^{1},\theta_{s}k^{2},\theta_{s}k^{n}\right\}$. The maximum charging utility at one stopping point is shown in Equation (16).

(16) $$U_{max}\left(s_{k}\right)=max\{U\left(s_{k},\theta_{s}k^{1}\right),U\left(s_{k},\theta_{s}k^{2}\right),\ldots ,U\left(s_{k},\theta_{s}k^{n}\right)\}$$

Here, $U\left(s_{k},\theta_{s}k^{n}\right)$ denotes the coverage area that is covered at the one-stop of MC in any MC antenna orientation $\theta_{sk}$. From different nodes, we obtain the link $L_{s}=\{l_{1},l_{2},\ldots ,l_{sn}\}$. We have to choose different stops of mobile charger $p_{k}=\{p_{1},p_{2},\ldots ,p_{n}\}$ to connect nodes for data transmission and their corresponding charging direction, where $\theta_{s}k^{1}\epsilon \{\theta_{s}k^{1}\theta_{s}k^{2},\ldots ,\theta_{s}k^{n}\}$. θ is the possible MC direction to transmit energy to different nodes. We use $U_{max}\left(s\right)$ to denote the maximum coverage area of the network with the help of MC from one stopping point of MC, shown in Equations (17)–(19).

(17) $$U_{max}\left(sk\right)\Sigma_{k-1}^{m}U_{max}\left(s\right)$$

(18) $$L_{s}=\{l_{s1},l_{s2},\ldots ,p_{sn}\},\left(sk\right)\epsilon cs$$

(19) $$\theta_{sk}^{1}\epsilon \{\theta_{sk}^{1}\theta_{sk}^{2},\ldots ,\theta_{sk}^{nk}\},\theta_{sk}^{1}\epsilon \{\theta_{sk}^{1},\ldots ,\theta_{sk}^{nk}\}$$

There is a different orientation of MC change to transmit energy to the different sensor nodes. We can calculate the possible coverage utilities at $s_{1}s_{2},\ldots ,s_{n}$ with one point of MC.

### 7.1. Algorithm for Length Constrained of Mobile Charger

In this section, we selected the tour length of MC for energy transmission. MC charges all the nodes which are in the coverage area. If some nodes that are very far from the MC tour cannot find energy, then MC selects these nodes in the second tour. So, we need to bind each MC tour to transmit maximum energy within a maximum time. In this section, first, we select a close energy transmission tour C for a set of selected sensor nodes $B\left(v_{k}\right)$ to maximize the accumulative charging utility gain. Then, we check the length C of the mobile charging tour. If yes, then C is a feasible energy transmission tour. A sensor node belonging to vϵc with minimum ratio $g\left(v_{k}\right)$ will be removed; $P\left(v_{k}\right)$ is defined as shown in Equation (20).

(20) $$\begin{array}{ll} p\left(v_{k}\right) & =\frac{g\left(v_{k}\right)}{B\left(v_{k}\right)}\\ & =\frac{\;\Sigma_{u\epsilon n_{c}+\left(v_{k}\right)}n_{c}+\left(v_{j}\right)\left(f\left(C_{u}\right)-f\left(RE_{u}\right)\right)}{\;\Sigma_{u\epsilon n_{c}+\left(v_{k}\right)}n_{c}+\left(v_{j}\right)\frac{c_{u}-re_{u}}{\eta }} \end{array}$$

Assume a sensor node v is removed from the MC charging tour C and also charged in the next charging tour. Two nodes are neighbor nodes in tour C, u and v, respectively. The existing two edges $\left(u,v\right)$ are in C. When node i is removed, both edges will also be removed which connects node i to node j and a new edge $\left(u,v\right)$ is added to the MC tour. The length C of MC will be reduced. The node removal procedure will continue until all the nodes receive proper energy from the MC.

### 7.2. Joint Base Algorithm

To tackle the problem, we proposed the following solution: we designed the joint-based algorithm for maximum energy transmission from DC and MC jointly to each sensor node. The only constraint comes from the limited energy that in each time slot, the total energy used by sensing and communication should never exceed the sum of the remaining energy and charged energy. The consumed energy should be expressed as

$$\phi^{\left(t\right)}={po}^{r_{i}^{t}}+pr\sum_{j=1}^{N_{\omega }^{\left(t\right)}}f_{ji}^{\left(t\right)}+\sum_{j=1}^{N_{\omega }^{\left(t\right)}}\left(pt+{\epsilon d}_{ij}^{4}\right){f\left(t\right)}_{ij}+\sum_{k=1}^{M_{s}}(pt+\epsilon_{ij}^{4})f_{in}^{\left(t\right)}$$

where po is the unit of energy of sensing and $0\leq \Phi_{i}^{\left(t\right)}\leq \Phi_{i}^{\left(t\right)}$. For each sensor node in state R, the total received energy can be expressed by

$$\Psi {\left(t\right)}_{i}=\sum_{m=1}^{M_{c}}\frac{a}{d_{im}+b}$$

where $0\leq \Psi {\left(t\right)}_{i}\leq \Psi_{max}$.

The remaining energy after time t can be calculated as

$$e_{i}^{\left(t\right)}e^{\left(t-1\right)}-\Phi {\left(t\right)}_{i}+\Psi {\left(t\right)}_{i}$$

Thus, the battery capacity constraints are obtained

$$0\leq e_{i}^{\left(t\right)}\leq B_{o}$$

Maximum energy transmission can help to increase the sensing rate of each node. The selection of a node in each set with both chargers depends on each node’s remaining energy, so we need to transmit maximum energy to each sensor node. At each time slot, after the selection of the optimal charging point $p_{i}$ in the set $s_{i}$, the MC antenna selects the node for energy transmission. MC should be covered with a specific network area, but the DC should cover the remaining area of RWSNs. The energy demand of each node should decide the angle of static DC. At each time slot, each node requires a different energy level. We set the score for each node in each iteration to evaluate its demand for being charged. Intuitively, the node with lower energy should have charging priority. On the other hand, we adopt a tree-based routing protocol in our design. Then, nodes in the upper layer should obtain more energy since they will take on more relay tasks.

Therefore, we designed such a scoring mechanism $\frac{g}{e+p+\varepsilon }$, where g is related to the number of node layers (a closer node is in a top layer), e denotes the remaining energy after the previous time slot, p is the charging power that the node has received in the current time slot, and ε is a factor to avoid the denominator being zero. Thus, we run the following mechanism in each time slot to obtain MC and DC orientation scheduling: in each iteration, we update each node’s score, then calculate each sector’s total score, and select the sector with the highest score greedily. This process continues until every charger has decided on its covering sector. After the charger’s orientation has been decided, obtaining each node’s energy level in the current time slot is easy. After that, we adopt the tree-based routing protocol to transmit data. In the network area, throughput is recorded, and the node’s energy is updated from time to time.

In a tree-based routing algorithm at each time slot, we need to check the data transmission rate to decide the energy transmission rate of each node. MC and DC jointly satisfy energy transmission. After that, the candidate’s next hope node with the smallest weight is selected as the next hope node while considering the route and finding. The charging capability can be expressed as the energy level, and the charging duration is equivalent to the size of the remaining energy and the charged energy. In the first layer, we consider the nodes with low battery energy and high energy consumption as dying nodes, or, more precisely, these nodes are more likely to die from exhaustion. In a network, the lower the residual energy, the greater the node’s energy consumption.

## 8. Performance Evaluation

In this section, we describe comprehensive simulation experiments to investigate the algorithm performance under different influence factors, such as sensing maximum data and transmitting data from each node. No study focuses on MC and DC joint energy transmission in the network area in the existing literature. In this paper, all the results are obtained from MATLAB.

### Simulation Setting

We consider RWSNs in which sensor nodes are equipped with the Powercast MC and DC and receivers. The 200 sensor nodes are randomly deployed in a 200 m×200 m area and are used to monitor the environment and charge the sensor node. In RWSNs, different base stations are used to analyze data from each set of network areas. In our proposed network area, we performed experiments with different numbers of requested nodes in which charging planning was arranged. The entire charging protocol was evaluated in real-time on-demand scenarios.

In RWSNs, we use DC and MC jointly for maximum energy transmission. Mobile Robots are used as the Mobile Charges (MC). To assess the effectiveness of our proposed scheduling policies, we will now present the performance evaluation in RWSNs, which is carried out using a discrete evolution simulator. To evaluate the merits of our proposed algorithm, we ran simulations to find the results. To understand the performance of RWSNs, the joint energy transmission level of MC and DC should be changed at each time slot to check the performance of each node. We mainly focused on the validity of our proposed algorithm with joint energy transmission protocol. The MC routing path should be increased and decreased according to the energy demand of the sensor node. The sensing rate of each node is randomly chosen. Each sensor node has a different energy consumption rate. MC randomly chooses each set of nodes. The corresponding information of the sensor node is generated and transmitted to the sink nodes.

In RWSNs, nodes send charging requests to DC and MC when their energy level is low. At each network set, MC records the energy request from each node, which is deployed in the DC coverage area as well as out of the coverage area of DC. The MC antenna changes its orientation at each time slot to transmit energy to RWSNs. The results show that DC transmits energy to each sensor node in the network area when sensor nodes send energy transmission requests. Recharging requests from each sensor node randomly arrived at chargers. MC selects its route to transmit energy to each sensor node. We used mixed integral programming to find the central point of MC to transmit maximum charging power to each sensor node.

**Figure 4:**
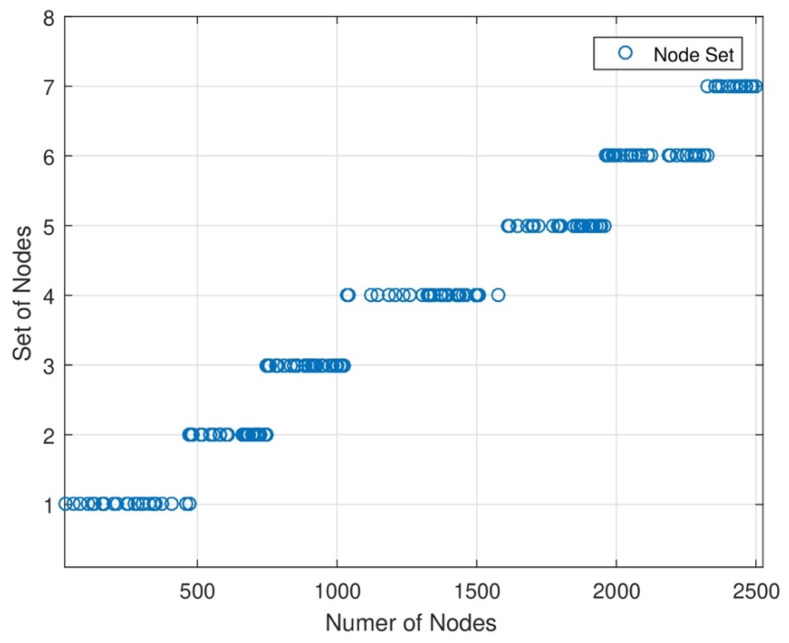
Impact of hop count on tour length.

In Figure 4, the network area is divided into subnetworks. Since different subnetworks and base stations are needed for data analysis, different nodes are located in each set of nodes in each subnetwork when designing the strategy. We can increase or decrease the length of each set of network areas. When the length of nodes in each set increases and decreases, the energy consumption level will change. In this figure, the energy consumption level is directly proportional to the data sensing rate of each node.

**Figure 5:**
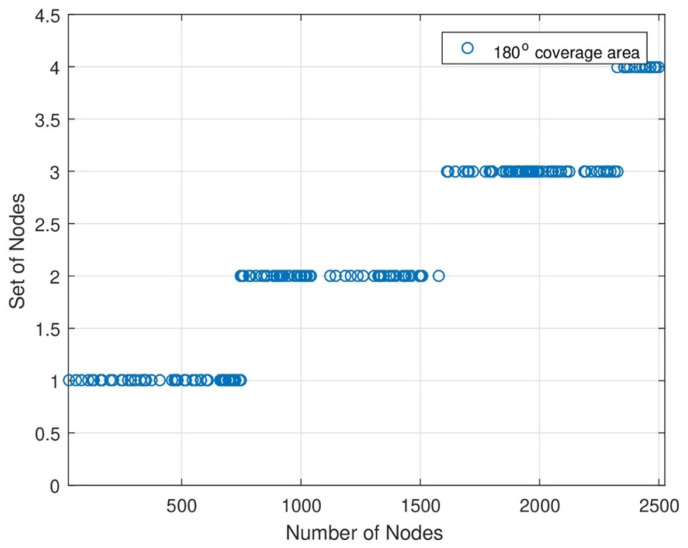
Set of nodes with $180^{\circ }$ coverage area.

Figure 5 shows that the network coverage area is 180°. In this graph, the DC covers several nodes for energy transmission. DC changes its orientation to transmit energy to each sensor node. When the number of nodes increases, the energy rate also increases. The remaining area of DC will be covered with MC for energy transmission. With the help of joint energy transmission, each sensor node consumes maximum energy from MC and DC. In each set of networks, we see each node’s data transmission percentage. The energy transmission level should also increase when the number of nodes increases. The novelty of this work is that the data transmission level is still maintained because of the MC and DC joint energy transmission.

**Figure 6:**
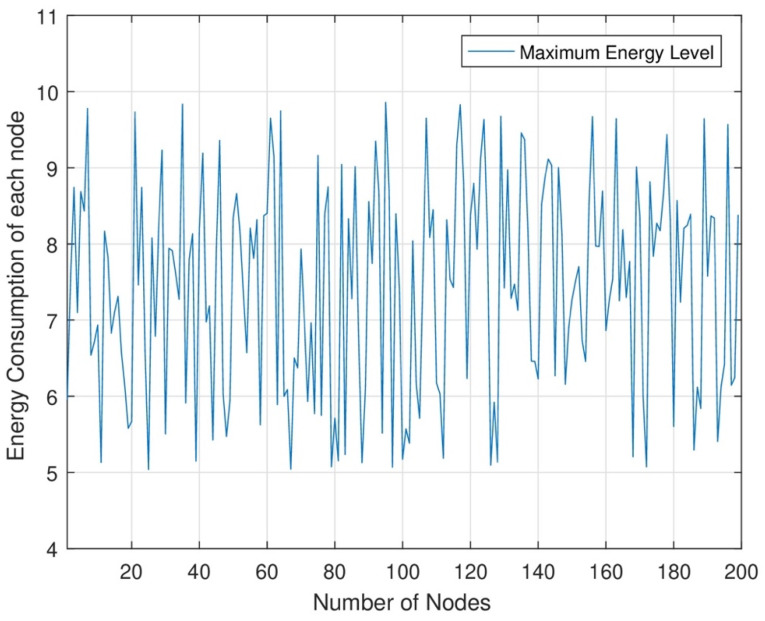
Different energy consumption levels of each node.

In Figure 6, the energy level of each node at different time slots is shown. In RWSNs, DC and MC jointly cover each set of network areas for maximum energy transmission to each sensor node. In RWSNs, we change the time slot allocation at each set of nodes for maximum energy transmission. As a result, each node consumes maximum energy from each node at each time slot. We can improve the network efficiency after transmitting maximum energy to each node.

**Figure 7:**
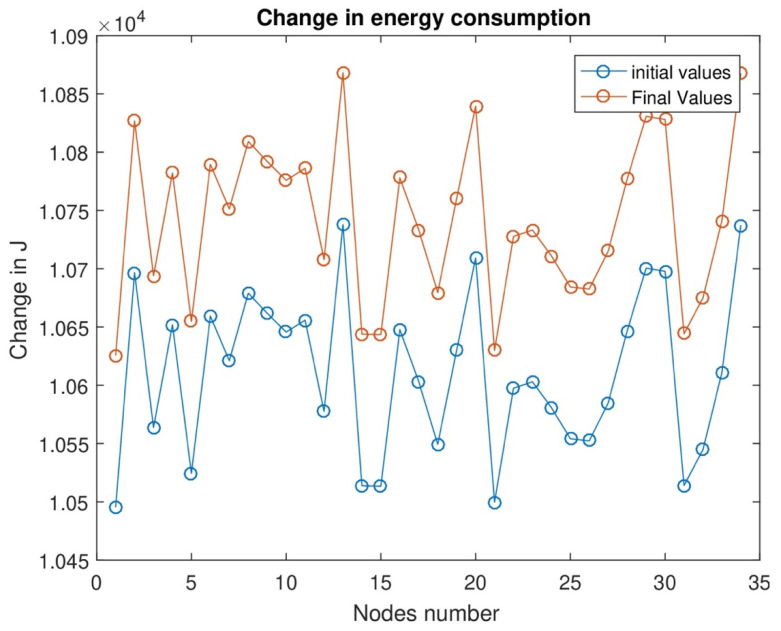
Energy consumption rate from initial to final level.

Figure 7 shows the set charging range. It is shown initially that the energy level rises and then declines with an increase. At the segment and final points, the whole charging system possesses the highest efficiency. In the subnetwork, each node is deployed in different sets. First, each node’s residual energy is more in the network area, indicating that the network works perpetually. However, simultaneously, in each set of networks, the residual energy of each node will reach maximum. This situation indicates that the maximum energy would be transmitted in each node. But at the same time, the node that consumes low energy would inevitably obtain more energy with the help of DC and MC. In the maximum energy transmission phenomenon, all the nodes in each set can harvest maximum energy from MC and DC.

**Figure 8:**
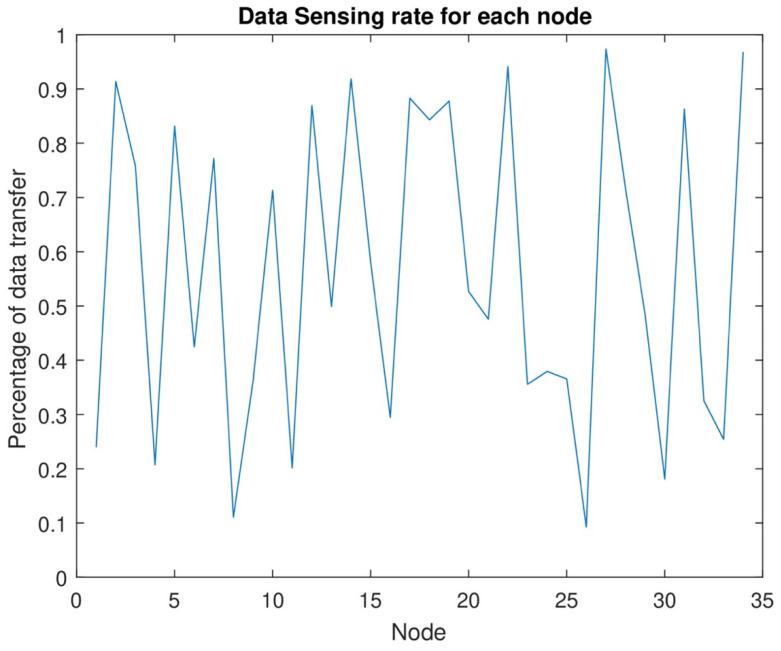
Data sensing rate of different nodes.

Figure 8 shows that the energy consumption of each node is the same as the data transmission level change at each time slot. In this graph, the data transmission level of each node is the same as the number of node changes in each set of networks. MC shows the energy transmission level of each node. When MC changes its charging cycle orientation, the energy level of each node is still maintained. At each round, nodes have different energy levels. When the energy level of each node is low, sensor nodes send charge requests to the MC. The MC comes in the set of networks to transmit energy to each set of nodes.

**Figure 9:**
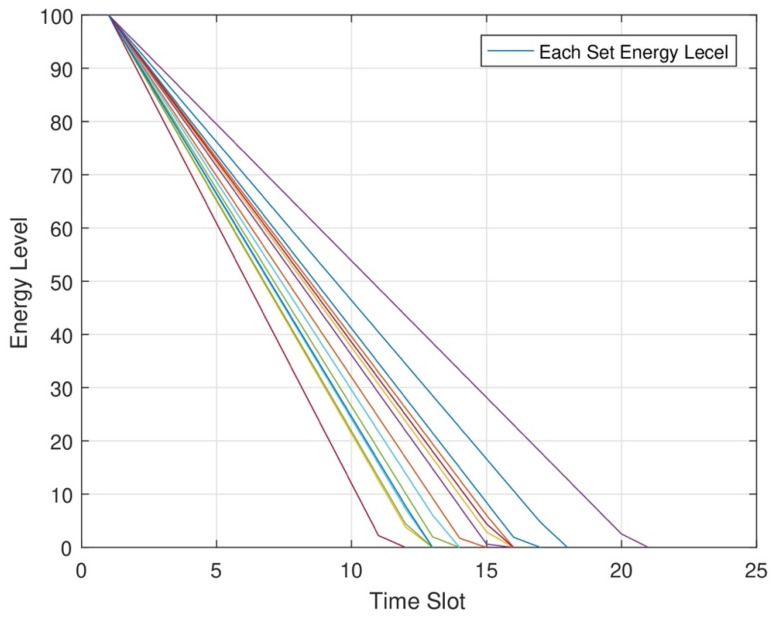
Maximum energy transmission in the maximum timeslot.

In Figure 9, we divide energy consumption into two stages: one is the initial level, and the other is the final level. The energy consumption level is low initially, and the data sensing level is low. In the final stage, we see that the energy consumption level increases as data consumption and data transmission levels increase.

**Figure 10:**
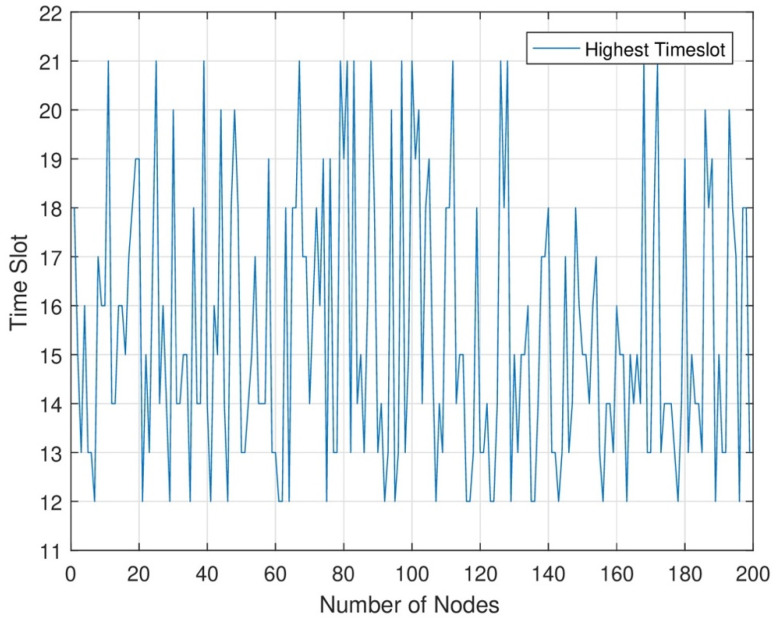
Highest energy transmission in the highest timeslot.

In Figure 10, in each time slot, when the number of nodes increases, the data sensing rate and data transmission rate increase. In the network area, when energy transmission is maximum in each node, we can increase each node’s time slot. When the time slot increases, the data sensing and transmission rate is maximum. In RWSNs, we see maximum time slot allocation. As a result, we can see that in each set of networks, we allocate a maximum time slot to sense maximum data from each sensor node.

In RWSNs, the node stops working as its residual energy is below its threshold level. Therefore, the lifetime ends. It can be seen that the residual energy of each node is still high, especially for the nodes with lower consumption. Without an energy supply, the network cannot work; it only runs for a minimum time. Only MC charging scheduling does not maintain the functionality of RWSNs perpetually. All the nodes do not gain energy before a specific level of energy. We need to replenish energy to maximize each node before its threshold level. In this regard, we used MC and DC jointly to transmit energy properly to each node and ensure that the network functions perpetually. It is clearly shown that our proposed joint energy transmission algorithm supports more nodes for maximum energy transmission.

**Figure 11:**
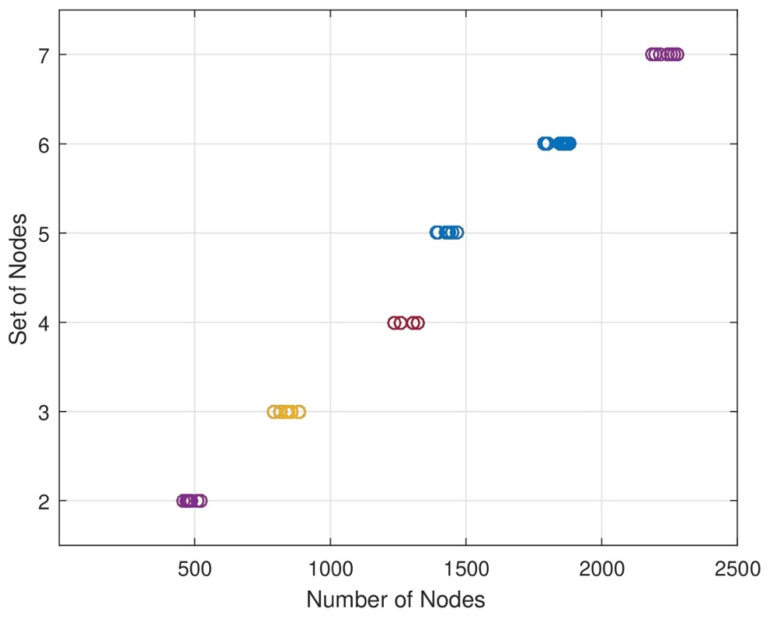
Node coverage area with $90^{\circ }$ angle.

Figure 11 shows the data sensing rate from the lowest rate to the highest sensing rate. The data sensing rate starts from the lowest level. The energy consumption rate also increases as the number of nodes increases. After the consumption of maximum energy, each node can sense maximum data. In this figure, the data sensing rate starts from zero when the energy level is near the dead level. When the energy consumption increases, the data sensing and data transmission rate should be high at each time slot. By the joint energy transmission of each node, the data sensing rate should be increased when the number of nodes increases. The network area has no data loss because of the highest energy transmission from MC and DC.

**Figure 12:**
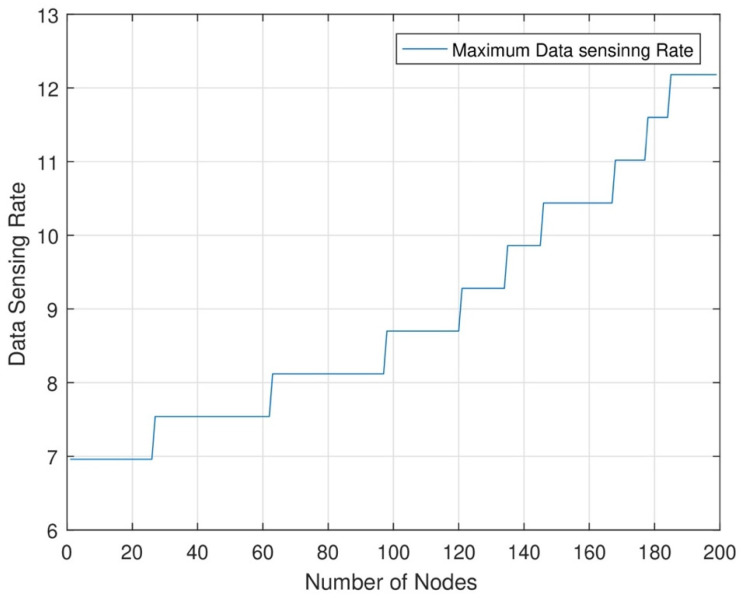
Data sensing rate from lowest to highest.

Figure 12 shows the maximum sensing rate of each node in each set. In this network, each node senses maximum data and transmits maximum data after transmitting maximum energy to each node. Each node can sense maximum data after the transmission of maximum energy. As a result, the figure shows each node’s highest data sensing rate. In this research, we can increase network efficiency with the help of maximum energy transmission from MC and DC and maximum data transmission.

Figure 12 illustrates that the maximum data sensing rate for nodes increases with an increase in the number of nodes. The maximum data sensing rate is essential for practical applications that necessitate timely and precise information. The information may be utilized for safety and decision-making purposes, among others. Examples of real-world applications encompass environmental monitoring, security surveillance, healthcare and patient data management, and early warning sensors. In these situations, ensuring that RWSNs function at full performance following efficient energy replenishment is crucial to sustaining high data throughput and network dependability.

**Figure 13:**
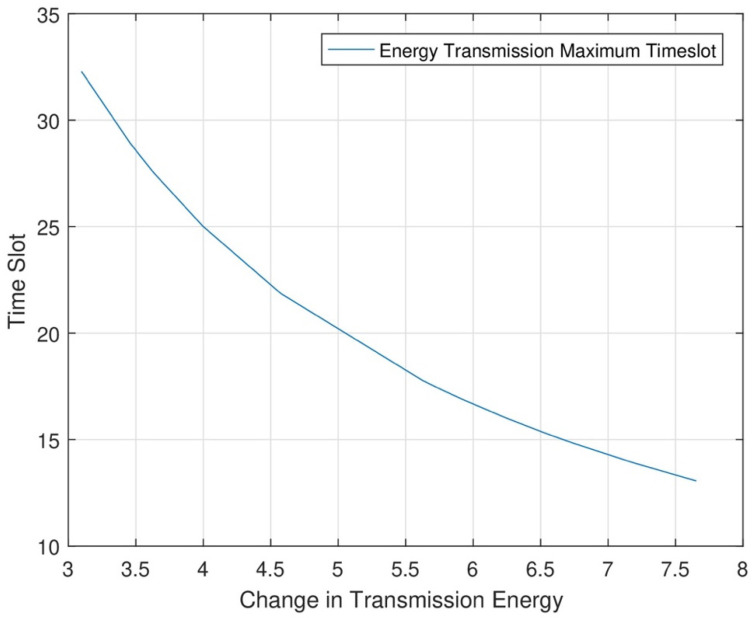
Energy consumption rate from highest to lowest.

Figure 13 shows the highest energy consumption rate to the lowest energy rate. As the timeslot and number of nodes increase at each set of nodes, the energy level decreases. The energy level decreases when the number of nodes increases in the network area. The efficiency of our proposed research is that each node maintains its working power to sense data from each node. Our proposed research shows that each node senses maximum data at each timeslot with the maximum energy transmission.

**Figure 14:**
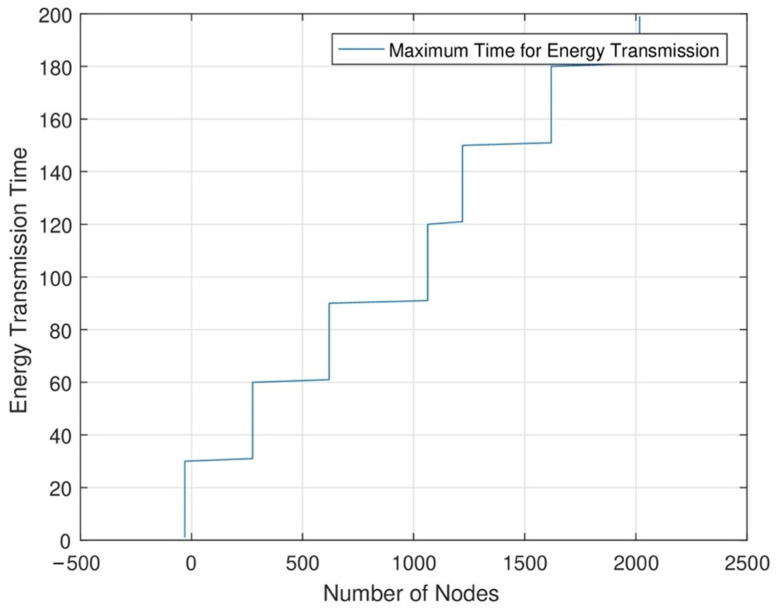
Impact of hop count on tour length.

Figure 14 shows the minimum slot length of each node. In RWSNs, the slot length is higher when data transmission and data sensing rates are high. When MC travels to find different sets from different distances for energy transmission, our proposed work’s performance increases. As a result, sensor nodes must be charged more frequently when MC and DC work together for energy transmission. MC and DC transmit energy to each node when both chargers send charging requests to each node.

## 9. Conclusions

In this research, we have studied the optimization charging path problem for rechargeable wireless sensor networks. To obtain a reasonable mobile charging route for the sensor node, we formulated the problem to sense maximum data from the area of the network. Our mobile charging schedule routing path can ensure the efficient work of RWSNs under certain constraint conditions. Meanwhile, it can maximize the network lifetime, which helps sense the maximum data from the area of RWSNs. We used the TSP algorithm, which helps transmit maximum energy to each sensor node fully. The algorithm works very effectively to transmit energy to each node. After the transmission of maximum energy to each node, all the nodes can easily sense data from the area of the network.

It is important to acknowledge the limitations of this study. The study uses a tree topology with fixed routes, which has several drawbacks, including scalability and single-point failure, which results in networking partition and congestion. Furthermore, it also lacks fault tolerance and load balancing.

## Figures and Tables

**Table 1:** Notation description.

Symbol	Definition
$r_{i}$	sensing rate of node i
n	number of nodes that are deployed in the area of the network
m	number of chargers that are deployed in the area of the network
$f_{it}^{\left(in\right)}$	data inflow in node i
$f_{it}^{\left(out\right)}$	data outflow of node i
$x_{i}j$	denote whether node i is in charger j coverage area
$e_{o}\left(i\right)$	parameter energy consumption for a node to sense one bit of data
$e_{r}\left(i\right)$	parameter energy consumption for a node to receive one bit of data
$e_{t}\left(i\right)$	parameter energy consumption for a node to transmit one bit of data
$e_{c}\left(ij\right)$	charging power that comes from charger j to node i
$x_{ij}$	node i coverage area where mobile charger j provides energy at each super node
$c_{ij}$	relationship between mobile charger and super node
$Y_{j}$	is the first super node where mobile charger j transmits energy to super node i
$P_{c}\left(i\right)$	position of the node where mobile charger j provides energy to the node i

## Data Availability

The data used in this study is not available as it was generated using simulations.
